# Estimation Model of Soil Freeze-Thaw Erosion in Silingco Watershed Wetland of Northern Tibet

**DOI:** 10.1155/2013/636521

**Published:** 2013-07-10

**Authors:** Bo Kong, Huan Yu

**Affiliations:** ^1^Chinese Academy of Sciences of Mountain Hazards and Environment, Chengdu 610041, China; ^2^College of Earth Sciences, Chengdu University of Technology, Chengdu 610059, China

## Abstract

The freeze-thaw (FT) erosion is a type of soil erosion like water erosion and wind erosion. Limited by many factors, the grading evaluation of soil FT erosion quantities is not well studied. Based on the comprehensive analysis of the evaluation indices of soil FT erosion, we for the first time utilized the sensitivity of microwave remote sensing technology to soil moisture for identification of FT state. We established an estimation model suitable to evaluate the soil FT erosion quantity in Silingco watershed wetland of Northern Tibet using weighted summation method of six impact factors including the annual FT cycle days, average diurnal FT phase-changed water content, average annual precipitation, slope, aspect, and vegetation coverage. Finally, with the support of GIS, we classified soil FT erosion quantity in Silingco watershed wetland. The results showed that soil FT erosion are distributed in broad areas of Silingco watershed wetland. Different soil FT erosions with different intensities have evidently different spatial and geographical distributions.

## 1. Introduction 

The annual freeze-thaw cycle of soils in cold regions is an important aspect of agricultural and ecological environments because this cycle may significantly impact soil physical properties [1, 2]. On the hydrological side, the precipitation occurring in early spring often results in high rates of runoff and erosion on frozen soil due to its poor infiltration capacity. Information about the depth of frost penetration in the subsurface is essential to avoid damage to roadbeds, structural foundations, sewers, and pipelines [3]. Soil freeze-thaw (FT) erosion is the gravity caused removing, migrating, and accumulating process of soil or rocks destructed mechanically by the differential expansion and contraction of different minerals induced by volume alteration of water at different phase due to temperature changes [4]. The main reason is that the moisture transfers to the freezing front during the freezing period, which results in water content increasing in places of shallow slope, and then frost heaving occurs under subzero temperature. The association and arrangement among soil particles was changed by frost heaving, and then the mechanical properties of the soil changed [5]. In the melting period of spring, the frozen layers in shallow slope thawed influenced by kinds of factors such as precipitation and increasing temperature. The melting water was hampered by unfrozen layer under them during their infiltration downward, results in the water content increasing rapidly in the layer between melt layer and frozen layer, and reaching saturation or super saturation state, then the effective stress within the slope reduced, the partial or whole of the shallow slope slide down along the water saturation layer under gravity [6]. It occurs mostly in the cold regions with high latitude and high altitude [7]. Recently with the onset of global warming there has been an increasing concern of greenhouse gas release from permafrost, and therefore interest in monitoring freeze-thaw erosion dynamics is increasing [8]. 

Soil freezing creates a situation where water and ice coexist in a near thermodynamic equilibrium. When soil temperature gradually drops below the freezing point, a portion of water in the soil turns into ice. The remainder of the water exists as adsorbed films around soil particles, in pores with sufficiently small diameters and in crevices between soil particles [9]. As the temperature continues to fall, more water becomes frozen, leaving residual liquid water in progressively thinner adsorbed films, smaller pores, and crevices. Consequently, a small amount of soil water may remain in a liquid state at temperatures well below the freezing point of water [10, 11]. In addition, an upward migration of soil water takes place as the frost penetrates the ground surface [12], a consequence of the thermal gradient causing capillary flow from higher (deeper soil) to lower temperatures. 

Present studies mainly focus on the impact of freeze-thaw erosion on the soil bulk density, permeability, moisture content, and stability [13–16], but about, estimation model of freeze-thaw erosion in large scale are still insufficient. The FT model includes six factors, annual FT cycle days, average diurnal phase-changed water content, annual average precipitation, slope and vegetation coverage has interest characteristics under the complex effects of freeze-thaw cycles, that factors take into account the environmental impact of soil properties, rainfall, topography, and ecology. Annual FT cycle days referred to freeze-thaw frequency; the freeze-thaw frequency at a particular station is the annual number of times the recorded temperature falls below the point of effective freeze following a period when the temperature was at or above the point of effective thaw. Phase change of soil water is an important sign of the development of soil freeze-thaw process causing the changing of liquid water content of soil. Wegmüller measured the brightness temperature change during several freeze/thaw circles using ground-based microwave radiometer and established a semiempirical model of frozen soil microwave emission [17]. Zuerndorfer developed a freeze-thaw state classification algorithm based on radiobrightness data from the Nimbus-7 Scanning Multichannel Microwave Radiometer (SMMR) [18, 19]. Other three factors were measured by the FT standard, respectively, from climate, topography, and vegetation growth status. Because there are few applications of FT estimate in the high altitude frozen region of China, not much work is done on the impact of freeze-thaw cycle on alpine areas.

Freeze-thaw area of more than 1,269,800 km^2^ accounted for about 13.4% of the entire China according to second national soil erosion data by remote sensing survey. The majority of the freeze-thaw erosion area is mainly distributed in Northeast, Northwest of high mountainous, and Tibetan Plateau. According to a survey, Tibetan Plateau, the main source of the Yangtze River and Yellow River, has FT erosion area of 1.04 × 10^6^ km^2^, which has seriously affected the production activity and life of local people and development of regional economy. Meanwhile [20, 21], the products of FT erosion have become the main source of sediment in the Yangtze River and Yellow River. Previous studies have also shown that FT erosion can increase soil erodibility and slop soil instability, thereby increasing the amount of soil loss by water [22–24]. In some FT erosion areas, the snowmelt runoff erosion at spring accounts for most of annual soil erosion. Therefore, accelerating researches on FT erosion and finding the effective methods and means to prevent FT erosion are extremely urgent and necessary. As water conservation functions of Silingco wetland has highly soil moisture and large temperature difference between day and night, it is located in the high-altitude of the Tibetan Plateau, its freeze-thaw erosion occurred significantly. In this paper, we established an estimation model of FT erosion and applied it for analysis of FT erosion in Silingco watershed wetland as an example.

## 2. Materials and Methods 

### 2.1. Study Area

**Figure 1:**
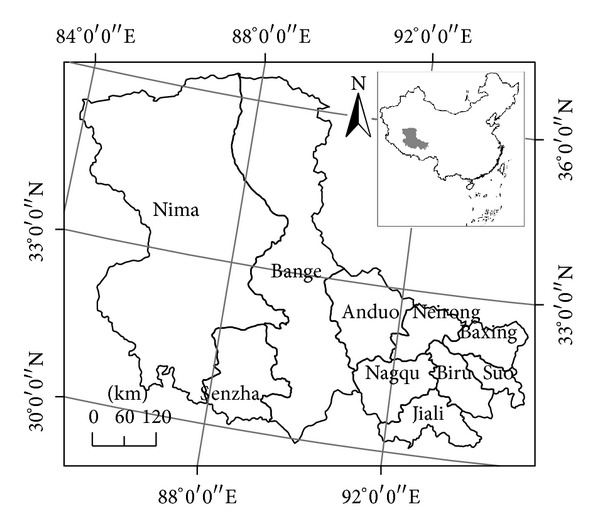
Spatial location of Silingco watershed wetland.

Silingco watershed wetland locates in Tibet Autonomous Region, bordering the northeastern Tibetan Plateau, the southern Xigaze, Lhasa and Linzhi, eastern Ali, western Ali, and the northern Xinjiang and Qinghai. It has total area of 39.46 km^2^, longitude of E 83°52′20′′~95°01′00′′, and latitude of N 29°56′20′′~36°41′00′′ (Figure 1). It has nine counties including Mani, Bange, Shenzha, Nagqu, Anduo, Suoxian, Baqing, Biru, and Jiali. The area has high altitude and low temperature [25]. The soil erosion in some regions is mainly FT erosion, which has appeared to have more and more evident influences on people's life and local economic development. 

### 2.2. Index Calculation

Estimation of the quantities of FT erosion should be based on the amount of the soil loss per unit area per unit time in the FT region. Currently, methods used to measure the quantities of FT erosion have not been reported in China and abroad; thus it is very difficult to quantitatively estimate the quantity of FT erosion. In fact, FT erosion intensity or the quantity of FT erosion differs in different FT erosion zones due to the different impacts of FT process, migration conditions of FT products, and the climatic factors. Therefore, we chose six most influential factors on FT occurrence and development including annual FT cycle days, average diurnal FT phase-changed water content, average annual precipitation, slope, aspect, and vegetation coverage to comprehensively evaluate the quantity of FT erosion. 

#### 2.2.1. Annual FT Cycle Days

The annual FT cycle day is the number of days that FT cycles occur in a year. We for the first time applied the microwave remote sensing technology for judging FT state. The land surface brightness temperatures at day and night were calculated using the AMSR-E (*The Advanced Microwave Scanning Radiometer*—*EOS*) data from satellite-borne passive microwave radiometer and used to calculate the criterion factors for measuring the changes in land surface temperature and emissivity. AMSR-E is modified from the Advanced Earth Observing Satellite-II in NASA. It observes atmospheric parameters, including precipitation, snow water equivalent, surface wetness, atmospheric cloud water, and water vapor. It seems worthwhile to examine the importance of freezing-thaw by investigating the frequency of freeze-thaw cycles by the use of AMSR-E. The discriminant analysis algorithm was used to classify the FT state at the land surface, label the regions with FT cycles, and statistically calculate the annual FT cycle day [26, 27]. The images were then used to generate a 30 m × 30 m grid map and the classification map of annual FT cycle day in the erosion areas. The FT criterion indexes are calculated as follows:

(1) $$\begin{array}{c} F=1.47{\text{Tb}}_{36.5V}+\frac{91.69{\text{Tb}}_{18.7H}}{{\text{Tb}}_{36.5V}}-226.77,\\ T=1.55{\text{Tb}}_{36.5V}+\frac{86.339{\text{Tb}}_{18.7H}}{{\text{Tb}}_{36.5V}}-242.41, \end{array}$$

where Tb is the polarized brightness temperature, 36.5*V* is the *V*-polarized brightness temperature of channel 36.5 GHz, and 18.7*H* is the *H*-polarized brightness temperature of channel 18.7 GHz. *F* > *T* indicates frozen soil, and otherwise, indicates thawed soil.

#### 2.2.2. Average Diurnal Phase-Changed Water Content

In the FT regions, the phase-changed water content reflects the difference in soil liquid water content during the FT process. The changes in potential land surface emissivity were extracted from AMSR-E data and used to estimate phase-changed water content at the land surface. Microwave remote sensing has potential ability to monitor the soil freeze-thaw process and accompanying water phase change. Ground experiments and model simulation of the characteristics of microwave radiation of soil freeze-thaw process constitute the base of microwave detection of soil freeze/thaw process. Soil freezing process is essentially a phase change process of liquid water. The microwave emission of soil is sensitive to soil liquid water content mainly because of the large difference of the dielectric constant between liquid water and dry soil and ice. The total phase-changed water content was accumulated based on the defined FT erosion zone and FT cycle days and used to calculate the average diurnal phase-changed water content [28–33] using the following formula:

(2) $$\begin{array}{c} m_{\text{pcv}}=A\;\left(\frac{{\text{Tb}}_{d,10.65H}}{{\text{Tb}}_{d,36.5H}}\;-\frac{{\text{Tb}}_{a,10.65H}}{{\text{Tb}}_{a,36.5H}}\right)\;+B, \end{array}$$

where *m*_pcv_ is phase-changed water content, *A* and *B* are the regression coefficients, subscript *d* indicates down rail, and subscript *a* indicates a rise track.

#### 2.2.3. Annual Average Precipitation

TRMM (*Tropical Rainfall Measuring Mission*) 3B42 jointly developed by US NASA and Japanese NASDA was used to measure the tropical rainfall. The system has three resolutions of 3 hours, 1 day, and 1 month. The monthly data was used in this paper. The average annual precipitation was calculated based on the monthly precipitation during 1998–2010.

#### 2.2.4. Slope and Aspect

A 30 m × 30 m grid slope and aspect data were generated using ArcGIS software based on the DEM data, which was obtained jointly by US NASA and NIMA at 30 m using SRTM1 because of its higher spatial resolution. 

#### 2.2.5. Vegetation Coverage

The vegetation coverage was converted from the data obtained by a NDVI (*Normalized Difference Vegetation Index*) MOD13Q1 instrument, which is a product of MODIS with resolution of 250 m and 16 days, based on the following formula:

(3) $$\begin{array}{c} \text{FVC}={\left(\frac{\left(\text{NDVI}-{\text{NDVI}}_{\min}\right)}{\left({\text{NDVI}}_{\max}-{\text{NDVI}}_{\min}\right)}\right)}^{K}, \end{array}$$

where FVC is the fractional vegetation coverage, NDVI is the normalized vegetation index, NDVI_max⁡_ is the maximum value of pure vegetation, NDVI_min⁡_ is the minimum value of pure bare soil, and *K* is an experience factor and its value is 1 in this paper. The resampling resolution of vegetation coverage is 30 m.

#### 2.2.6. Estimation Model of FT Erosion Content

**Figure 2:**
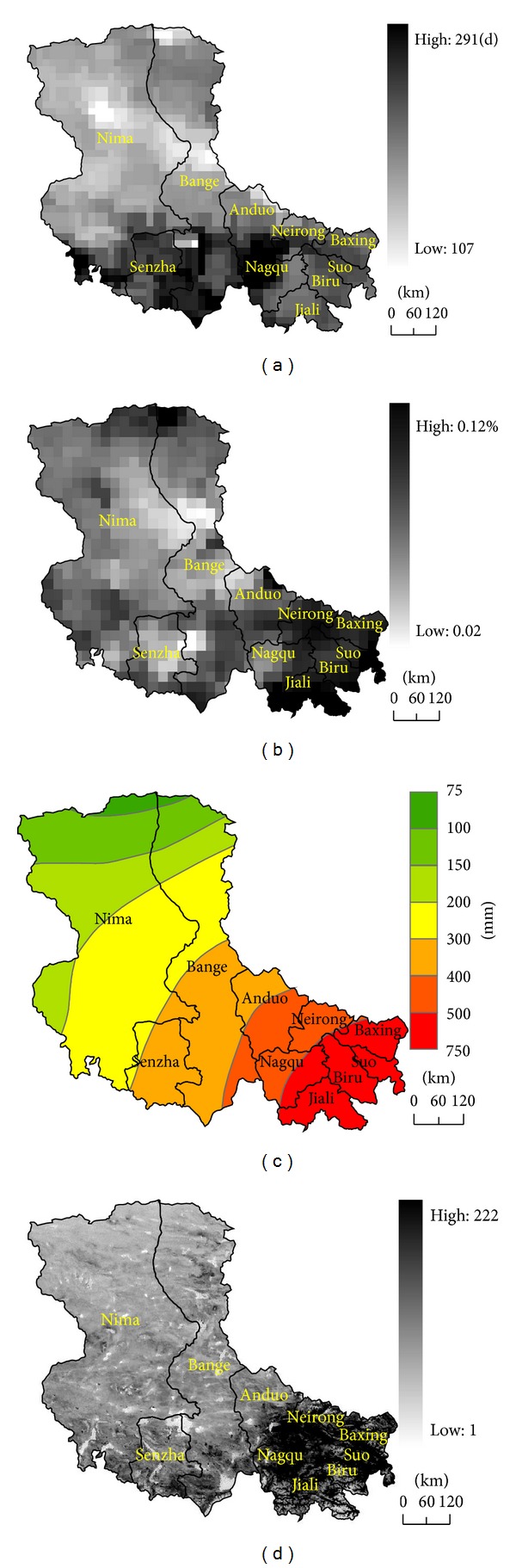
Distribution maps of annual FT cycle days (a), average diurnal phase-changed water content (b), average annual precipitation (c), and vegetation coverage (d).

The comprehensive evaluation of FT erosion quantity is a complex process of a number of affecting factors to make it an integrated single index. In fact, the evaluation classification is only possible in one dimension. This requires integration of multiple factors impacting the FT erosion to obtain a comprehensive evaluation index. In this paper, we adapted a weighted summation method to calculate the comprehensive evaluation index using the following formula:

(4) $$\begin{array}{c} I=\frac{\sum_{i=1}^{n}W_{i}I_{i}}{\sum_{i=1}^{n}W_{i}}, \end{array}$$

where *I* is the dimensionless comprehensive evaluation index of FT erosion corresponding to erosion intensity, *n* is the six indicators, *W*_*i*_ is the weight of each index, and *I*_*i*_ is the dimensionless value of each index in different ranges. The weights of classification factors for FT erosion were determined by using analytic hierarchy process. First, the six factors (annual FT cycle days, average diurnal phase-changed water content, average annual precipitation, slope, aspect and vegetation coverage (Figure 2)) were pairwise compared and used to build a comparison matrix. Then their weights and consistency were calculated and tested using the square root method.  Table 1 lists the weight values of the six factors.

## 3. Results

### 3.1. Classification Map and Analysis of Each Indicator

By introducing the indexes, we obtained the annual FT cycle days and the average diurnal phase-changed water content in eight years from 2003 to 2010, the average annual precipitation, slope, and aspect with 30-meter resolution in 12 years from 1998 to 2010, and the average fractional vegetation coverage from 2003 to 2010 (Figure 1).  Table 1 lists the assigned classification value of each evaluation factor.

As shown, the annual FT cycle day did not have similar distribution pattern with diurnal phase-changed water content. Shenzha County and Anduo County in south Silingco watershed wetland had higher FT cycle day while Neirong, Biru, Jiali, and Suoxian in the east Silingco watershed wetland had higher diurnal phase-changed water content. The 12-year average annual precipitation increased mainly from west to east. The areas with relatively flat terrain, abundant rainfall, and relatively low altitude in the east Silingco watershed wetland had higher vegetation coverage.

### 3.2. Estimation of the Quantity of FT Erosion

**Figure 3:**
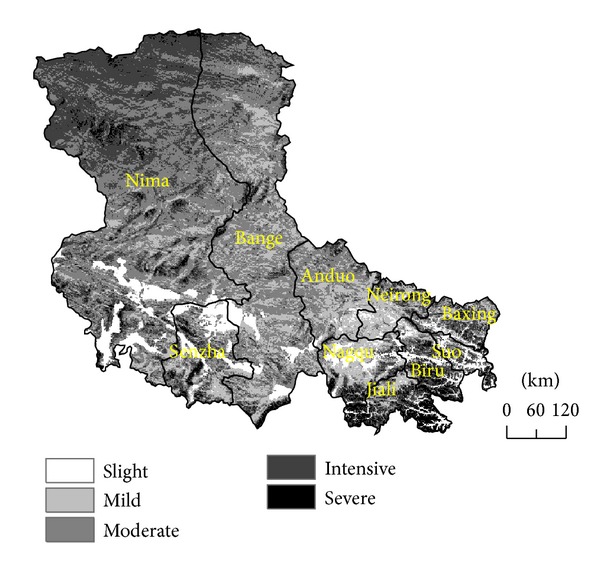
Classification map of the quantity of freeze-thaw erosion in Silingco watershed wetland.

Based on the erosion index calculated from formula (4), the FT erosion in Silingco watershed wetland could be divided into 6 categories: no erosion (*I* < 1), slight erosion (1 < *I* < 2.2), mild erosion (2.2 < *I* < 2.5), moderate erosion (2.5 < *I* < 2.8), intensive erosion (2.8 < *I* < 3.3), and severe erosion (3.3 < *I* < 4). The index standard distribution of erosion zones in Silingco watershed wetland is shown in Figure 3.

Erosion is widely distributed in Silingco watershed wetland of Northern Tibet, with FT erosion intensity mainly in moderate and intensive categories. The total FT erosion area in the region is 3.91 × 10^5^ km^2^. Among them, the slight, mild, moderate, intensive, and severe erosion areas are 3.07 × 10^4^ km^2^, 6.89 × 10^4^ km^2^, 1.7 × 10^5^ km^2^, 1.06 × 10^5^ km^2^, and 1.55 × 10^4^ km^2^, accounting for 7.8%, 17.62%, 43.47%, 27.1% and 3.9% of the total erosion area, respectively.

### 3.3. The Spatial Distribution of FT Erosion

The spatial distribution of the different FT erosion zones differed significantly. The slight erosion zone was concentrated in the southwest areas and southeast river of Silingco watershed wetland (Figure 3) and distributed in the low-lying potential of lakes, rivers, and wetlands. Its rainfall is between 200 and 300 mm, slope was less than 3°, and aspect was less than 45°. In addition, the area has high vegetation coverage and the lowest spatial position of average diurnal phase-changed water content. For example, Gerencuo basin in Shenzha county, Jiali basin, and Suoxian basin had less FT erosion. Mild FT erosion was distributed mainly in the south of Silingco watershed wetland and located in the transition areas of the slight and moderate erosion zones, such as Nagqu, Neirong, and Anduo. These areas had rainfall between 300 and 500 mm and higher degree of vegetation coverage. The moderate FT erosion zones accounted for the most majority and were distributed mainly along Silingco watershed wetland. These areas had high incidence of avalanche, relatively high elevation, and different rainfall from 75 mm to 750 mm. The intensive FT erosion zones accounted for the second most majority and were distributed mainly in northern Silingco watershed wetland. These areas were uninhabited and had a greater degree of natural disasters and quite different average annual temperature. The severe FT erosion zones were surrounded by the intensive erosion zones and included Jiali, Biru, and north of Nimaxian. These areas had minimum proportion and distributed mostly around the watershed.

### 3.4. Accuracy Assessment

To test the accuracy of the calculated freeze-thaw erosion rank, we adopted the basic error matrix and precision index in the field survey. A total of 252 points were sampled with different spatial location, land use types, fractional vegetation cover change, landforms, aspect, slope, and erosion types, and so on, and analyzed using the principal component analysis to obtain the optimum index and weights.  Table 2 lists the error matrix of the calculated erosion category and the field survey.

 The map precision proved the effectiveness of the classification method. The mild intensity erosion map reached 91% precision and the extreme intensity, high intensity, and middle intensity erosion maps reached 60% precision (Table 3), reflecting the high credibility of the classification results. The mild and extreme intensity erosion maps also had high preciousness, although the area distribution of sharp erosion was low. The overall precision indicated that the classification results were in agreement with the actual erosion level. Their probability reached 76.2%, illustrating that the freeze-thaw erosion model of Silingco watershed wetland is effective and accurate.

## 4. Discussions and Conclusions

FT erosion, as one of the of major erosion types in China, is mainly located in the western China and has not yet attracted enough attention in China and abroad. Studies on FT erosion are rare and far behind those on the water and wind erosion. In particular, usually occurred freeze-thaw erosion research of Plateau wetlands is insufficient.

Previous models for FT erosion estimation are not very accurate and lack scientific basis due to poor selection of impact factors, such as temperature, slope, aspect, vegetation coverage, annual precipitation, and soil. In this paper, we, from the scientific point of view, introduced microwave remote sensing techniques to determine the state of FT erosion and monitor the FT process, which allow us attribute FT erosion to the development of surface permafrost and parameters monitoring. By using the microwave remote sensing techniques, we can detect the soil sensitivity to the changes of moisture and calculate the annual FT cycle days and average diurnal phase-changed water content. These selected factors are reasonable and scientific by the high erosion accuracy.

The FT erosion estimation model established in this paper has certain scientific basis and strong operationality and practicability. The results have certain significance for the in-depth understanding of the occurrence and development process of FT erosion in Silingco watershed wetland of Northern Tibet, and for the rational use and preservation of the ecological environment. Follow-up studies will further strengthen the mechanisms underlying the capacity of a typical FT erosion zone, the causes for the spatial distribution of moderate erosion area, the evaluation precision, and the ecological consequences of erosion.

## Figures and Tables

**Table 1:** Classification assignment of evaluation indices and their weights.

Index	Assignment criteria				Weight (*W*_*i*_)
Annual FT cycle days (day)	≤60	100–120	120–200	>200	0.15
Average diurnal phase-changed water content (%)	≤0.03	0.03–0.05	0.05–0.07	>0.07	0.15
Average annual precipitate (mm)	≤150	150–300	300–500	>500	0.05
Slope (°)	0–3	3–8	8–15	>15	0.35
Aspect (°)	0–45,	45–90,	90–135,	135–225	0.05
	315–360	270–315	225–270		
Vegetation coverage (%)	60–100	40–60	20–40	0–20	0.25
Assigned value	1	2	3	4	

**Table 2:** Error matrix of the quantity of FT erosion.

	FT erosion results being evaluated					
	Severe erosion	Intensive erosion	Moderate erosion	Mild erosion	Slight erosion	Sum
Level of sampling point						
Severe erosion	5	1	2	1	1	**10**
Intensive erosion	0	23	3	5	9	**40**
Moderate erosion	3	2	27	4	5	**41**
Mild erosion	2	0	4	16	6	**28**
Slight erosion	1	3	7	1	121	**133**
Sum	**11**	**29**	**43**	**27**	**142**	**252**

**Table 3:** Precision index of the quantity of FT erosion.

	Cartographic precision	Omission	User accuracy	Commission
Severe erosion	0.50	0.50	0.45	0.55
Intensive erosion	0.58	0.43	0.79	0.21
Moderate erosion	0.66	0.34	0.63	0.37
Mild erosion	0.57	0.43	0.59	0.41
Slight erosion	0.91	0.09	0.85	0.15

## References

[1] Qi J, Ma W, Song C (2008). Influence of freeze-thaw on engineering properties of a silty soil. *Cold Regions Science and Technology*.

[2] Viklander P (1998). Permeability and volume changes in till due to cyclic freeze/thaw. *Canadian Geotechnical Journal*.

[3] Eigenbrod KD (1996). Effects of cyclic freezing and thawing on volume changes and permeabilities of soft fine-grained soils. *Canadian Geotechnical Journal*.

[4] Tang KL (2003). *Soil and Water Conservation in China*.

[5] Shan W, Guo Y, Liu H (2009). Effect of freeze-thaw on strength and microstructure of silty clay. *Journal of Harbin Institute of Technology*.

[6] Niu F, Cheng G, Lai Y, Jin D (2004). Instability study on thaw slumping in permafrost regions of Qinghai-Tibet Plateau. *Chinese Journal of Geotechnical Engineering*.

[7] Tang KL (1999). Characteristics and perspectives on scientific discipline of soil erosion and soil and water conservation in China. *Research of Soil and Water Conservation*.

[8] Sun Y, Cheng Q, Xue X (2012). Determining in-situ soil freeze-thaw cycle dynamics using an access tube-based dielectric sensor. *Geoderma*.

[9] Bai JH, Gao HF, Xiao R (2012). A review of soil nitrogen mineralization as affected by water and salt in coastal wetlands: issues and methods. *Clean-Soil Air Water*.

[10] Spaans EJA, Baker JM (1995). Examining the use of time domain reflectometry for measuring liquid water content in frozen soil. *Water Resources Research*.

[11] Bai J, Deng W, Wang Q, Cui B, Ding Q (2007). Spatial distribution of inorganic nitrogen contents of marsh soils in a river floodplain with different flood frequencies from soil-defrozen period. *Environmental Monitoring and Assessment*.

[12] Lee J, Lee S (2002). The frost penetration with the modified soil in the landfill bottom liner system. *Geosciences Journal*.

[13] Kok H, McCool DK (1990). Quantifying freeze/thaw-induced variability of soil strength. *Transactions of the American Society of Agricultural Engineers*.

[14] Mostaghimi S, Young RA, Wilts AR, Kenimer AL (1988). Effects of frost action on soil aggregate stability. *Transactions of the American Society of Agricultural Engineers*.

[15] Chamberlain EJ, Gow AJ (1979). Effect of freezing and thawing on the permeability and structure of soils. *Engineering Geology*.

[16] Bai J, Cui B, Chen B (2011). Spatial distribution and ecological risk assessment of heavy metals in surface sediments from a typical plateau lake wetland, China. *Ecological Modelling*.

[17] Wegmüller U (1990). The effect of freezing and thawing on the microwave signatures of bare soil. *Remote Sensing of Environment*.

[18] Zuerndorfer B, England AW (1992). Radiobrightness decision criteria for freeze/thaw boundaries. *IEEE Transactions on Geoscience and Remote Sensing*.

[19] Zhang J, Mark AR, Jeffery G (2012). Discretization approach in integrated hydrologic model for surface and groundwater interaction. *Chinese Geographical Science*.

[20] Formanek GE, McCool DK, Papendick RI (1984). Freeze-thaw and consolidation effects on strength of a wet silt loam. *Transactions of the American Society of Agricultural Engineers*.

[21] Bai JH, Lu QQ, Wang JJ (2013). Landscape pattern evolution processes of alpine wetlands and their driving factors in the Zoige plateau of China. *Journal of Mountain Science*.

[22] Bai J, Ouyang H, Xiao R (2010). Spatial variability of soil carbon, nitrogen, and phosphorus content and storage in an alpine wetland in the Qinghai-Tibet Plateau, China. *Australian Journal of Soil Research*.

[23] Sharratt BS, Lindstrom MJ Laboratory simulation of erosion from a partially frozen soil.

[24] Sharratt BS, Lindstrom MJ, Benoit GR, Young RA, Wilts A (2002). Runoff and soil erosion during spring thaw in the northern U.S. Corn Belt. *Journal of Soil and Water Conservation*.

[25] Zhang JP, Zhang YL, Liu LS (2011). Predicting potential distribution of Tibetan spruce (Picea smithiana) in Qomolangma (Mount Everest) National Nature Preserve using maximum entropy niche-based model. *Chinese Geographical Science*.

[26] Williams RBG, Robinson DA (2001). Experimental frost weathering of sandstone by various combinations of salts. *Earth Surface Processes and Landforms*.

[27] Zuerndorfer BW, England AW, Dobson MC, Ulaby FT (1990). Mapping freeze/thaw boundaries with SMMR data. *Agricultural and Forest Meteorology*.

[28] Wang P, Jiang LM, Zhang LX (2011). Evaluation of the impact of lake ice on passive microwave snow retrieval algorithms using ground-based observation. *Remote Sensing Application*.

[29] Schmugge T, O’Neill PE, Wang JR (1986). Passive microwave soil moisture research. *IEEE Transactions on Geoscience and Remote Sensing*.

[30] Li SH, Demin DMZ, Luan ZQ (2011). Quantitative simulation on soil moisture contents of two typical vegetation communities in Sanjiang Plain, China. *Chinese Geographical Science*.

[31] Jackson TJ, Schmugge TJ (1989). Passive microwave remote sensing system for soil moisture: some supporting research. *IEEE Transactions on Geoscience and Remote Sensing*.

[32] Foster JL, Hall DK, Chang ATC, Rango A (1984). An overview of passive microwave snow research and results. *Reviews of Geophysics & Space Physics*.

[33] Chang ATC, Foster JL, Hall D (1987). Nimbus-7 SMMR derived global snow cover parameters. *Allnals of Glaciology*.

